# *Drosophila* PLP assembles pericentriolar clouds that promote centriole stability, cohesion and MT nucleation

**DOI:** 10.1371/journal.pgen.1007198

**Published:** 2018-02-09

**Authors:** Helio Roque, Saroj Saurya, Metta B. Pratt, Errin Johnson, Jordan W. Raff

**Affiliations:** The Sir William Dunn School of Pathology, University of Oxford, South Parks Road, Oxford, United Kingdom; Washington University School of Medicine, UNITED STATES

## Abstract

Pericentrin is a conserved centrosomal protein whose dysfunction has been linked to several human diseases. It has been implicated in many aspects of centrosome and cilia function, but its precise role is unclear. Here, we examine *Drosophila* Pericentrin-like-protein (PLP) function *in vivo* in tissues that form both centrosomes and cilia. *Plp* mutant centrioles exhibit four major defects: (1) They are short and have subtle structural abnormalities; (2) They disengage prematurely, and so overduplicate; (3) They organise fewer cytoplasmic MTs during interphase; (4) When forming cilia, they fail to establish and/or maintain a proper connection to the plasma membrane—although, surprisingly, they can still form an axoneme-like structure that can recruit transition zone (TZ) proteins. We show that PLP helps assemble “pericentriolar clouds” of electron-dense material that emanate from the central cartwheel spokes and spread outward to surround the mother centriole. We propose that the partial loss of these structures may largely explain the complex centriole, centrosome and cilium defects we observe in *Plp* mutant cells.

## Author summary

Centrioles are complex, microtubule (MT) based structures that organise two important cell organelles, the centrosome and the cilium. The centrosome is a major MT organising centre in many cell types, while the cilium functions as a cellular “antenna” responsible for regulating several cellular signalling pathways. Pericentrin is conserved centriole-binding protein that plays an important part in centrosome and cilium function, and mutations in the *Pericentrin* gene are linked to several human diseases. Here we use the fruit-fly *Drosophila melanogaster* to investigate how Pericentrin-Like-Protein (the fly homolog of Pericentrin) contributes to centriole, centrosome and cilium function. We find that *Plp* mutant fly centrioles have subtle structural defects, organize less microtubules, and do not properly migrate to the cell membrane to form cilia. We also observe that PLP helps assemble “pericentriolar clouds”—dense structures that emanate from the centriole, and appear to interact with microtubules, as well as connect existing centrioles to newly formed ones. In mutant flies these structures are significantly reduced in size. We propose that the defects in these PLP structures can explain most, if not all, the complex defects observed in *Plp* mutants.

## Introduction

Centrioles are complex MT-based structures that duplicate precisely once per cell cycle when a “daughter” centriole is assembled on the side of a “mother” centriole during S-phase [1]. In proliferating tissues, centrioles form centrosomes when they recruit pericentriolar material (PCM) around themselves [2]. The PCM contains several hundred proteins [3], including many involved in nucleating and organising MTs, and centrosomes function as major MT organising centres (MTOCs) in many eukaryotic cells. In interphase, the centrioles organise relatively small amounts of PCM and so organise relatively small numbers of MTs. As cells enter mitosis, the PCM expands dramatically (a process termed centrosome maturation) allowing the centrosomes to organise many more MTs, which is important for efficient cell division [2,4]. In non-proliferating tissues, the centrioles often migrate to the cell cortex, where the mother centriole organises the assembly of a cilium. The cilium can be motile—so moving the cell, or generating liquid flow around the cell—or immotile, with mechano- and/or chemo-sensory functions. Defects in centriole, centrosome and cilium function have been linked to a wide range of human pathologies, including cancer, microcephaly, dwarfism and obesity [5,6].

Pericentrin is one of the best studied centrosomal proteins, and it has been implicated in several aspects of centriole, centrosome and cilium function [7]. In vertebrates, Pericentrin is important for mitotic centrosome maturation [8–12], and it is also cleaved by Separase towards the end of mitosis, allowing mother and daughter centrioles to disengage from one another [13–15]. In flies, however, the Pericentrin-like-protein (PLP) appears to have a more minor role in centrosome maturation [16–18], while a role in regulating centriole disengagement has not been reported. Instead, studies in fly cultured cells suggest that PLP has a critical role in organising the interphase PCM [19]. During interphase, PLP molecules are organised around the mother centriole in a polarised manner, with their C-termini linked to the centriole and their N-termini extending outwards [19,20], and PLP helps recruit PCM proteins such as γ-tubulin and Cnn to the interphase centriole [19]. Pericentrin adopts a similarly polarised conformation around the interphase mother centriole in vertebrate cells [12,21], where it has also been implicated in cilia function [22] and the DNA Damage Response (DDR) [23,24].

Pericentrin defects have been linked to several human diseases [7]. Most prominently, mutations in human *Pericentrin* cause microcephalic osteodysplastic primordial dwarfism (MOPD) or Seckel syndrome, diseases associated with severe growth retardation during both foetal and post-foetal development [25–27]. Pericentrin dysfunction has also been linked to several other diseases such as mental disorders [28], and diabetes [29]. In none of these cases, however, is it understood how Pericentrin defects contribute to these complex pathologies.

Clearly it is important to understand Pericentrin function within the context of a developing organism, but such an analysis is complicated in vertebrates because centrosome and cilia defects lead to pleiotropic organismal defects. In vertebrates, the loss of centrosomes activates a p53-dependent pathway leading to cell death or senescence [30], while the loss of cilia leads to severe developmental defects [31]. *Drosophila* is an attractive model system for this type of study, as flies can proceed relatively normally through most of development in the absence of centrioles, centrosomes and cilia [32]. Centrosomes are essential, however, for the development of the early syncytial embryo [33–35], and elevated levels of apoptosis, and a high incidence of mitotic errors have been reported in some fly tissues when centrioles are lost or are defective [36], but not others [32,33,37]. Moreover, unsurprisingly, cells with defective centrosomes are more dependent on other pathways for spindle assembly, such as the spindle assembly checkpoint, and the augmin pathway of MT nucleation [36,38,39]—although centrosome loss does not appear to transcriptionally upregulate any of these pathways [40].

Here we examine *Drosophila* PLP function in the Sensory Organ Precursor (SOP) lineage of the pupal notum [41], and in the spermatocyte lineage of the testes—tissues in which the centrioles initially form centrosomes and then form cilia [42]. In the pupal notum, SOP cells initially form centrosomes as they progress through two rounds of asymmetric division to produce a sensory organ comprising four cells (bristle, socket, sheath and neuron). After division is complete, only the centriole pair in the neuron will go on to form a cilium, which is used to sense the mechanical movement of the bristle cell [41,43]. In the sperm lineage, a gonialoblast initially forms centrosomes and proceeds through 4 rounds of symmetric division to produce 16 primary spermatocytes. These cells dramatically grow in size during an extended G2 period, during which the centrioles also dramatically enlarge and form short cilia; the function of these cilia is currently unknown [42]. The primary spermatocytes then proceed through two rounds of meiotic division to generate a cyst of 64 spermatids.

Using a combination of live cell imaging, Electron Microscopy (EM) and Electron Tomography (ET) we show that PLP helps to organise electron-dense “pericentriolar clouds” around the mother centriole that extend outwards from the cartwheel spokes past the centriolar MTs. These clouds organise MTs around the centriole during interphase, and help connect the mother centriole to its engaged daughter. These clouds are greatly diminished in *Plp* mutants, and we propose that this may explain many of the pleiotropic centriole, centrosome and cilium defects we observe in *Plp* mutant cells.

## Results

### Centrioles separate prematurely, and are mis-positioned relative to the apical cortex in *Plp* mutant SOPs

**Fig 1:**
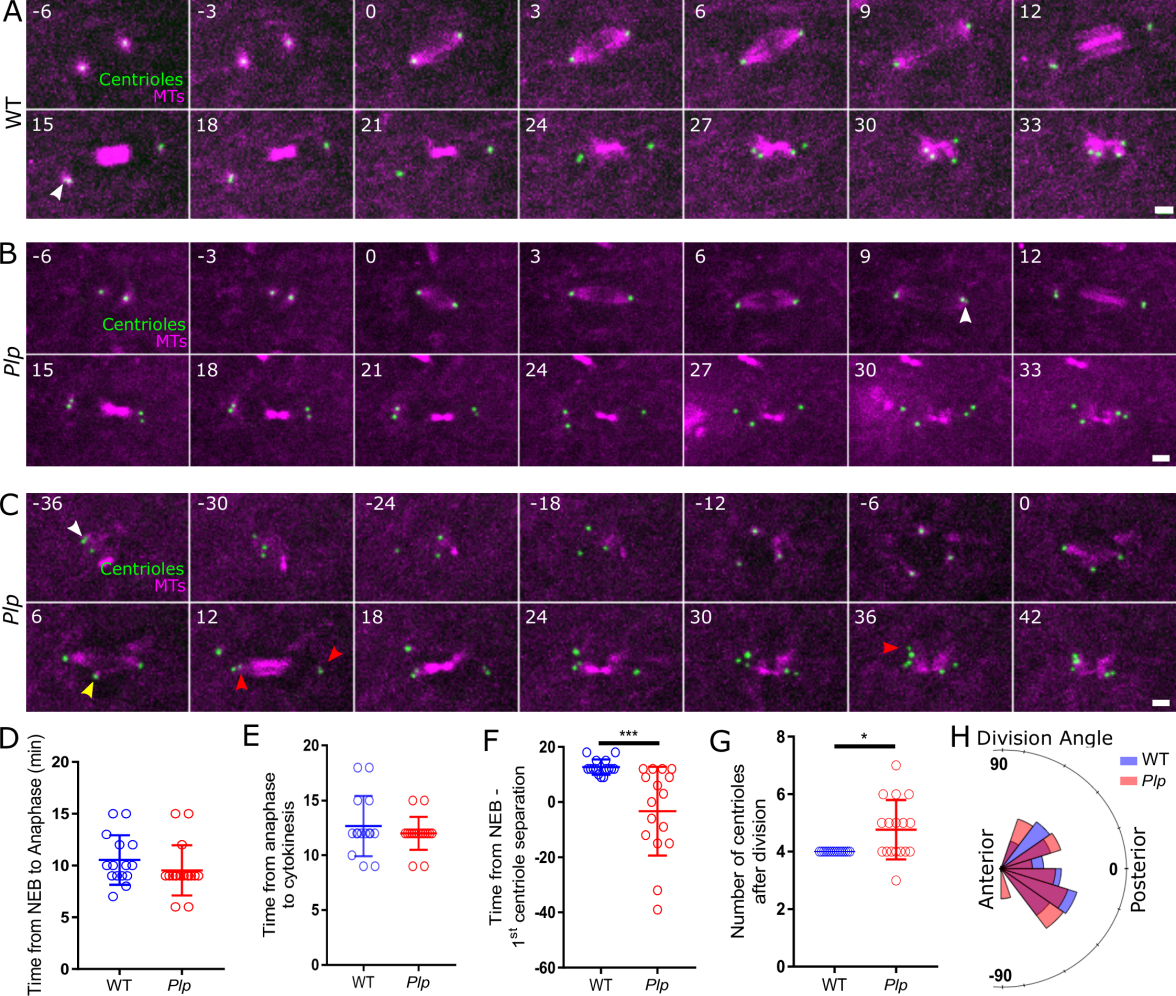
Mitosis is not dramatically perturbed in *Plp* mutant SOPs, but centrioles can separate prematurely. (**A-C**) Images from videos of living WT (A) or *Plp* mutant (B,C) SOPs expressing Jupiter-mCherry to reveal the MTs (*magenta*) and Asl to reveal the centrioles (*green*). Time in minutes relative to nuclear envelope breakdown (NEB) (t = 0) is indicated. *White* arrowheads indicate when centriole separation is first detected; *yellow* arrowheads indicate an extra spindle pole; *red* arrowheads indicate instances where the centrioles in cells with extra centrioles separate. (**D-G**) Graphs compare various aspects of the behaviour of WT (*blue*) and *Plp* mutant (*red*) cells, as indicated. (**H**) Chart shows the division angle relative to the anterior/posterior axis of WT and Plp mutant SOPs. Distributions were assessed by the D'Agostino & Pearson normality test. Significance test for normal distributions was made by an unpaired two-tailed T-Students test and for non-normal distribution by Mann-Whitney ranking test. Bars indicate the mean +/- the SD. All significance tests shown in this and subsequent Figures were performed and presented in this manner, unless specified. Information on numbers analysed and biological repeats for these and all other experiments is given in S1 Table. In (G) a Wilcoxon signed-rank test was used to compare the median of *Plp* mutant to the WT value of 4. Scale bar = 2μm (A-C). * p < 0.05, *** p < 0.001.

**Fig 2:**
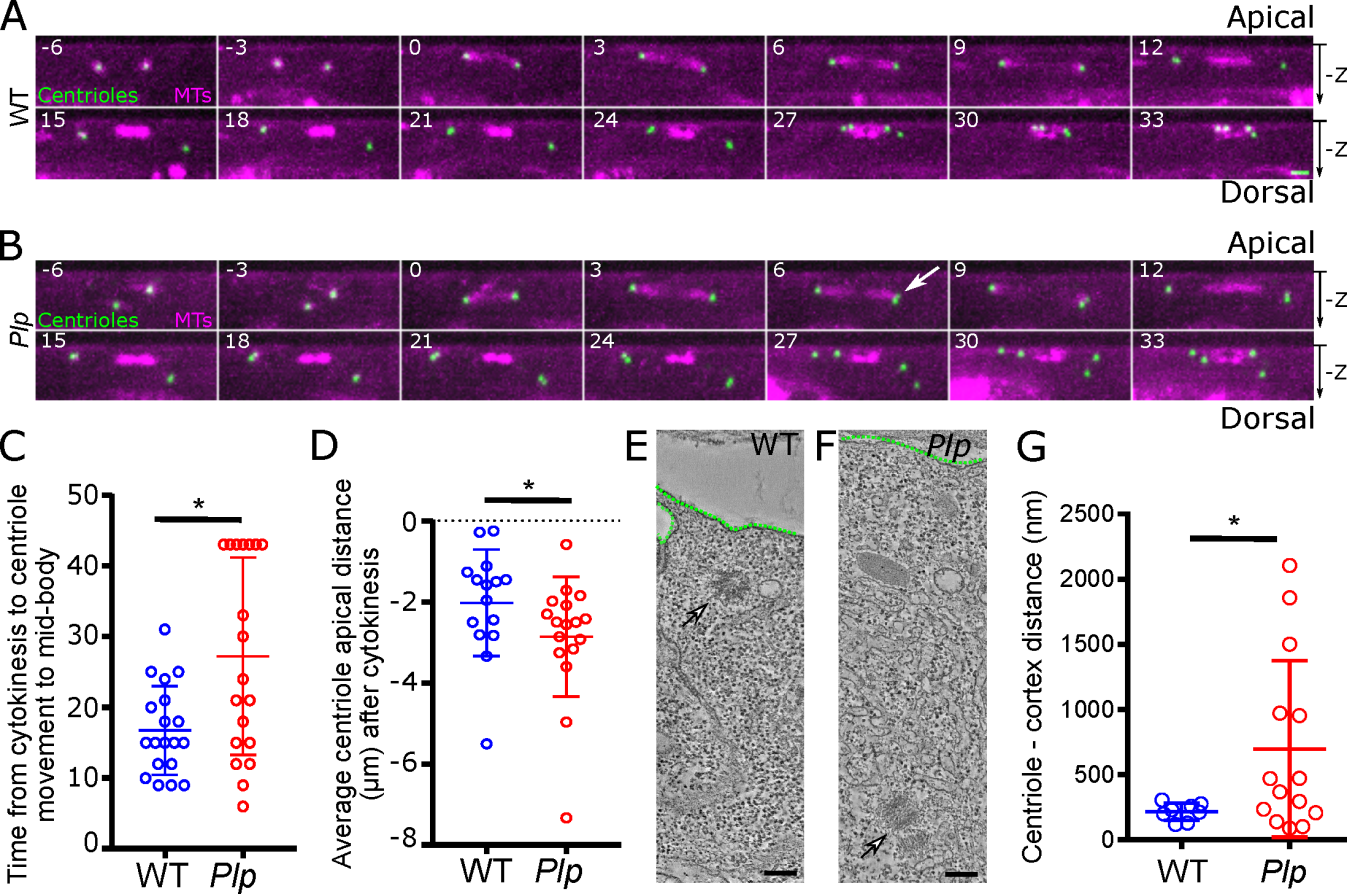
Centrioles are not positioned properly relative to the cortex in *Plp* mutant nota. (**A,B**) Images from videos of living WT (A) or *Plp* mutant (B) SOPs expressing Jupiter-mCherry to reveal the MTs (*magenta*) and Asl to reveal the centrioles (*green*). Time in minutes relative to nuclear envelope breakdown (NEB) (t = 0) is indicated. These images are taken from the same videos shown in Fig 1A and 1B, but shown from a side-on view to the spindle. *White* arrow indicates a centriole pair separating prematurely. (**C,D**) Graphs compare the behaviour of WT (*blue*) and *Plp* mutant (*red*) cells, as indicated. (**E,F**) Images from electron tomograms (ETs) of WT (E) or *Plp* mutant (F) pupal notum cells, highlighting the position of the centrioles (arrows) relative to the cell cortex (dotted *green* line). (**G**) Graph quantifies the centriole-to-cortex distance in pupal notum cells. Scale bar = 2μm (A, B) or 100 nm (E, F) * p < 0.05.

To gain a better understanding of the role of PLP during mitosis in flies we imaged the first cell division of SOP cells in the notum of either *WT* (Fig 1A) or *Plp* mutant pupae (see Materials and Methods) (Fig 1B). *Plp* mutant SOPs fell into two categories: (1) 10/17 SOPs (~59%) entered mitosis with two centrosomes but, in 5/10 of these cells, centriole separation occurred prematurely compared to WT (arrows, Fig 1A, t = 15 and Fig 1B, t = 9; Fig 1F); (2) 7/17 SOPs (41%) entered mitosis with centrioles that had already prematurely separated (arrow, Fig 1C, t = -36; Fig 1F). These cells formed multipolar spindles as they entered mitosis (*yellow* arrow, Fig 1C, t = 6), but became bipolar before anaphase onset (Fig 1C, t = 12). Many of the centrosomes that had separated prematurely appeared to duplicate (*red* arrows, Fig 1C, t = 12), so daughter cells often had too many centrosomes (*red* arrow, Fig 1C, t = 36; Fig 1G). *Plp* mutant SOPs were not delayed in mitosis (Fig 1D and 1E), and there were no detectable defect in spindle alignment relative to the anterior-posterior body axis (Fig 1H). Thus, cell division appears relatively unperturbed in *Plp* mutant SOPs, but centrioles can separate prematurely, leading to centriole amplification and spindle multipolarity. These defects do not appear to cause any developmental problems in the mutant flies, where centrosome amplification is surprisingly well tolerated [44].

When viewed along the apical-basal axis, the spindles in WT SOPs were well aligned with the cortex during metaphase—early-anaphase (Fig 2A, t = 6–12), but the posterior centriole pair moved basally during late-anaphase—telophase (t = 15-18mins), before moving apically again as the centrioles separated (t = 21–27)—as described previously [45]. At the end of mitosis all 4 centrioles were clustered at the cortex, close to the spindle mid-body-remnant (t = 30–33). In *Plp* mutant SOPs the spindles aligned with the cortex during metaphase—early anaphase (Fig 2B, t = 6–12), and the posterior centriole pair moved basally during late-anaphase—telophase (t = 15-18min), but the movement of the centrioles back to the apical cortex was erratic, and often delayed (Fig 2B, t = 21–33; Fig 2C). At the end of mitosis, the average distance between the centriole and the apical cortex was slightly increased in *Plp* mutant nota (Fig 2D). Moreover, an EM analysis revealed that while the centrioles in WT nota were usually clustered close to the apical cortex, they were often displaced basally in *Plp* mutant nota (Fig 2E–2G).

Taken together, these studies suggest that the centrioles in *Plp* mutant nota exhibit two major defects: (1) Mother and daughter centrioles separate prematurely either prior to mitosis (potentially allowing centrioles to overduplicate), or during mitosis; (2) The centrioles do not efficiently migrate to the apical cortex at the end of mitosis, and have difficulty in establishing and/or maintaining their normal positioning at the apical cortex.

### PLP helps to organise electron-dense “prericentriolar clouds” that surround the mother centriole

**Fig 3:**
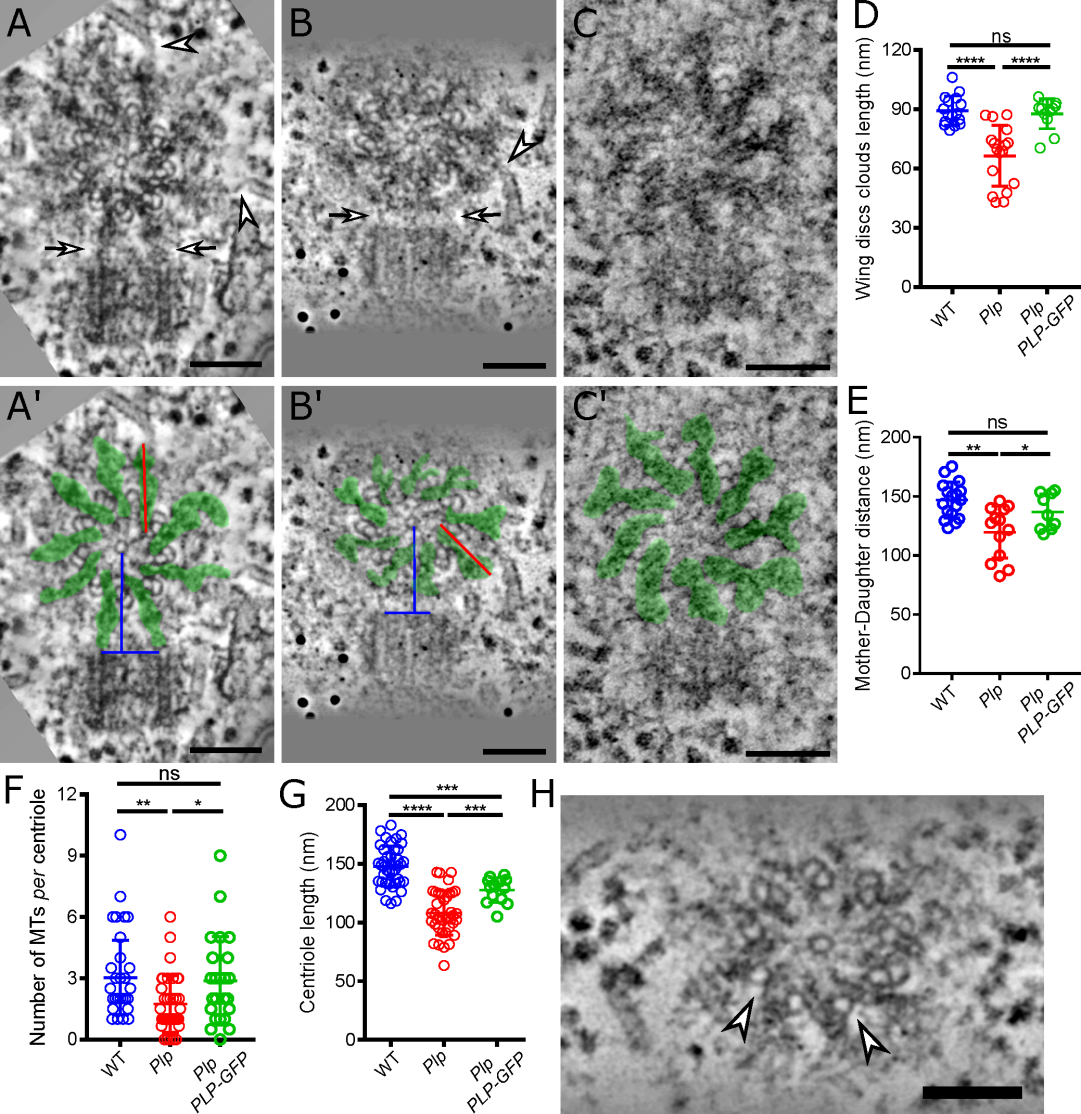
*Plp* mutants assemble reduced “pericentriolar clouds” around the mother centriole. (**A-C**) Images from electron tomograms (ETs) of WT (A), *Plp* mutant (B) or *Plp* mutant rescued by PLP-GFP (C) centrioles in wing disc cells. Cytoplasmic MTs contacting the centrioles are highlighted by arrowheads, and electron-dense connections between the mother and engaged daughter are highlighted with arrows. (A’-C’) Images are the same as in (A-C) but with the electron dense pericentriolar clouds highlighted in *green*, with *red lines* illustrating how cloud length is measured, and *blue lines* illustrating how the distance between the cartwheel of the mother centriole and the edge of its engaged daughter is measured. Note that, for unknown reasons, we consistently found it difficult to clearly visualise the centriole MTs in *Plp* mutant cells that were rescued by PLP-GFP (C,C’), although the pericentriolar clouds were very obvious. (**D-G**) Graphs compare various aspects of centriole structure and behaviour in WT (*blue*), *Plp* mutant (*red*), and *Plp* mutant rescued by PLP-GFP (*green*) cells, as indicated. (**H)** Electron tomogram image from a *Plp* mutant wing disc centriole; arrowheads highlight missing outer B-MTs. Scale bars = 100nm; * p < 0.05, ** p < 0.01, *** p < 0.001 **** p < 0.0001.

We examined *Plp* mutant centriole ultrastructure in more detail using EM and electron tomography (ET). The pupal notum has a thick cuticle making fixation difficult, and, although we could clearly detect centrioles in both the WT and mutant tissue (Fig 2E and 2F; S1A and S1B Fig) their morphology was usually poor. We therefore turned to 3^rd^ instar larval wing-discs—a tissue where centriole ultrastructure is better observed [46]. A striking feature of the tomograms of WT wing disc centrioles was the presence of “pericentriolar clouds” of electron dense material that surround the mother centriole: these clouds seemed to originate close to the outer spokes of the central cartwheel and extend outwards through the gaps between the MT blades (Fig 3A and 3A’). In *Plp* mutant centrioles, electron-dense clouds emanated from the cartwheel spokes and spread past the MT blades, but these were greatly diminished in size (Fig 3B, 3B’ and 3D). A similar phenotype was observed in *Plp* mutant notum centrioles, although the detailed ultrastructure of the centriole was difficult to discern (S1 Fig). Importantly, the transgenic expression a PLP-GFP fusion protein driven from the ubiquitin promoter [47] significantly rescued the pericentriolar cloud defect in mutant wing-disc centrioles (Fig 3C, 3C’ and 3D). We conclude that PLP is required for the proper assembly and/or maintenance of these pericentriolar clouds.

### The pericentriolar clouds appear to promote centriole engagement

We noticed that in WT centriole pairs, robust pericentriolar clouds emanating from the mother directly contacted the proximal end of the engaged daughter centriole (n = 17/17 centriole pairs examined) (arrows, Fig 3A). In *Plp* mutant centriole pairs (n = 13/13), the daughter was still engaged with the mother, but the size of the pericentriolar clouds contacting the daughter were greatly diminished (arrows, Fig 3A and 3B). As a result, the distance between the centre of the mother centriole and its engaged-daughter was significantly reduced in *Plp* mutant centriole pairs, a phenotype that was significantly rescued by the expression of PLP-GFP (Fig 3E). Although the mother and daughter centrioles are slightly closer together in *Plp* mutants, this does not appear to lead to an increase in the strength of engagement, as mutant mother and daughter centrioles are prone to disengage prematurely (Fig 1; Fig 2; see also below). Instead, our observations suggest that the pericentriolar clouds connecting the mother and daughter centriole (arrows, Fig 3A) normally help to promote engagement, potentially explaining why *Plp* mutant centriole pairs tend to disengage prematurely.

### The pericentriolar clouds help to organise interphase MTs

Previous studies have shown that PLP promotes the organisation of the interphase PCM in cultured *Drosophila* cells [19], and we noticed that MT ends were often closely associated with the pericentriolar clouds (arrowheads, Fig 3A and 3B). To test if PLP was involved in organising these MTs we counted the number of MTs within 100nm of the centriole MT wall. Significantly fewer MTs were associated with the centrioles in *Plp* mutant tissues when compared to WT, and this phenotype was significantly rescued by the expression of PLP-GFP (Fig 3F). We conclude that PLP helps organise interphase MTs around the centriole.

### Centrioles are too short and their organisation is subtly perturbed in the absence of PLP

Our EM analysis revealed two further aspects of the *Plp* mutant centriole phenotype. The centriole MT blades usually comprise a complete inner A-MT that shares part of its outer wall with an incomplete B-MT (in many organisms the B-MT shares part of its outer wall with another incomplete C-MT—so making a MT-triplet, but most fly centrioles normally contain MT doublets). In seven of the nineteen singlet or mother centrioles we observed in *Plp* mutant wing discs several of the centriolar MT doublets were missing the outer B-MT (arrows, Fig 3G)—something we have never observed in WT centrioles. This suggests that the PLP may help to assemble and/or stabilise the centriolar B-MTs. We also noticed that centrioles were significantly shorter in the *Plp* mutant tissues, although this phenotype appeared to only be partially rescued by the expression of PLP-GFP (Fig 3H). We conclude that centriole structure is subtly perturbed in *Plp* mutants.

### Plp mutant sensory cells lack cilia due to a failure to establish and/or maintain the position of the centrioles at the cell cortex

**Fig 4:**
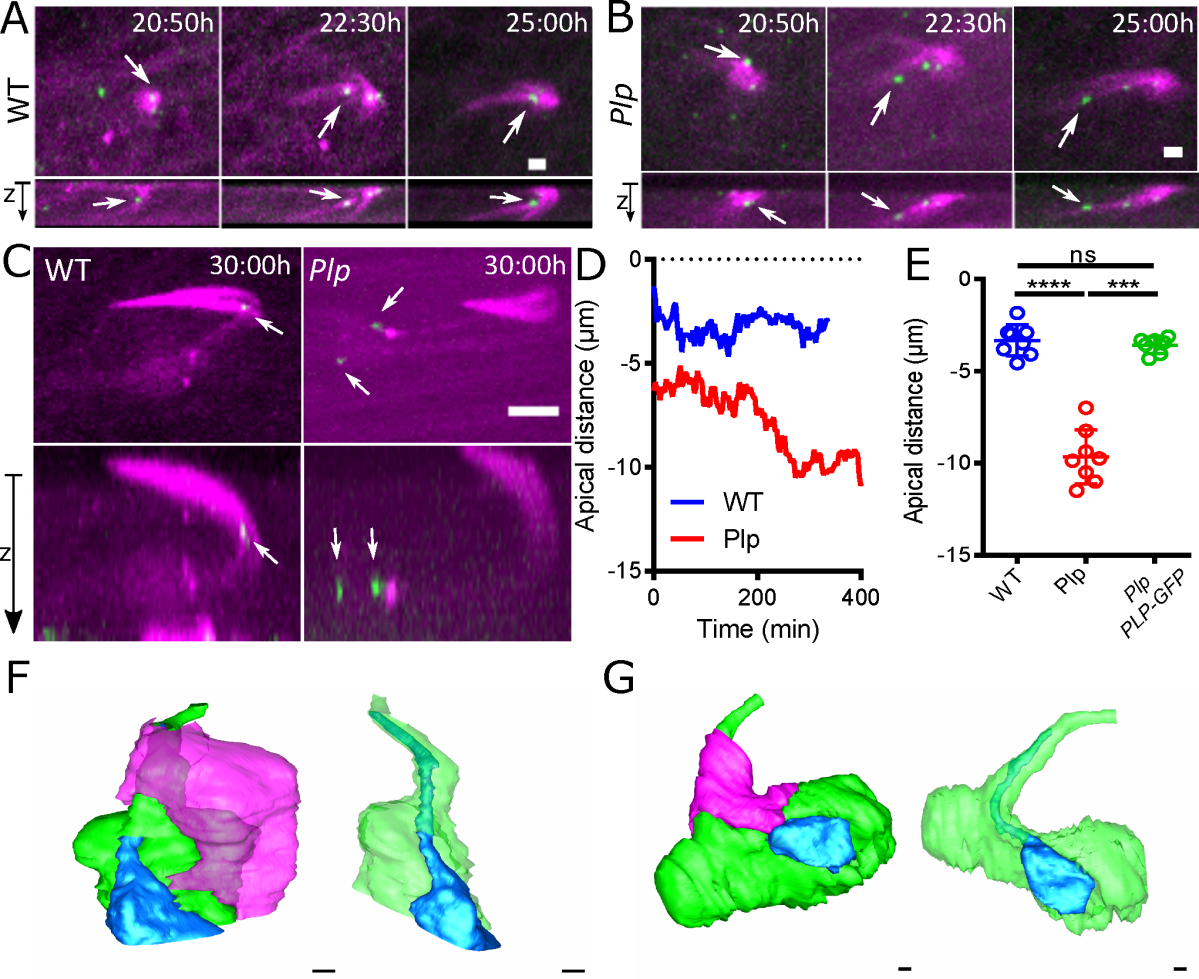
Basal bodies appear to be specified properly in *Plp* mutant sensory neurons, but they are mis-positioned. **(A-C)** Images from videos of living WT or *Plp* mutant sensory organs, as indicated, expressing Jupiter-mCherry to reveal the MTs (*magenta*) and Cep-104-GFP to reveal the centrioles and basal bodies (*green*). Time in hours:minutes after puparium formation (APF) (t = 0) is indicated. **(D)** Graph charts the position of the brightest Cep-104-GFP containing centriole (that will become the basal body) relative to the cortex in a single WT (*blue*) or *Plp* mutant (*red*) sensory organ, illustrating how the centriole appears to gradually drift away from the cortex. **(E)** Graph quantifies the distance of the brightest Cep-104-GFP containing centriole from the cortex at 30:00h AFP (when ciliogenesis is normally complete) in WT (*blue*), *Plp* mutant (*red*) or *Plp* mutant rescued by PLP-GFP (*green*) nota. (F,G) Images show 3D-reconstructions from SBF-SEM data of the cells in a WT (F) or *Plp* mutant (G) pupal notum sensory organ. The Sensory Neuron (*blue*) sends an extension (that would normally contain the axoneme close to its tip) through the cell body of the Bristle Cell (*green*); the Support Cell (*magenta*) is illustrated in the images, on the left, but not on the right, which are also rotated by 90^o^. The overall organisation of the *Plp* mutant organ is not detectably perturbed. Scale bar = 2μm (A, B) or 5μm (C) or 500 nm (F,G).

A lack of PLP/Pericentrin leads to defects in cilia function in flies and vertebrates [16,22], and our analyses above suggest that centrioles have difficulty in migrating to, and/or establishing/maintaining their position at, the apical cortex—which could lead to a failure in cilium assembly. To test this possibility, we analysed centriole behaviour in the developing pupal-notum, starting at 20:50h APF—when cell divisions in the SOP lineage are complete, but before the sensory cilia have started to form. We labelled centrioles with Cep104-GFP, a protein that localizes to both centrioles and cilia [48,49]. In WT organs a prominent array of MTs started to form at 20:50hr APF associated with the forming bristle cell; several centrioles were detectable at this stage, but the most brightly labelled centriole (that will eventually form the basal body) was consistently slightly displaced below the MT array (arrow, Fig 4A, 20:50hr APF). By 22:30hr APF, this centriole had established a sub-apical position close to the MT array of bristle cell, and this position was maintained during ciliogenesis (arrow Fig 4A, 22:30–25:00hr APF; Fig 4D).

In *Plp* mutant sensory organs the MT array associated with the bristle cell was formed, and a prominently labelled centriole was initially detectable close to it (arrow, Fig 4B 20:50hr APF), but this centriole failed to establish a stable sub-apical position relative to the MT array and it usually became further displaced from the cortex as development proceeded (arrow, Fig 4B, 22:30–25:00hr APF; Fig 4D). At 30hr APF, when cilia are forming, a single brightly labelled centriole pair remained positioned close to the MT array in WT sensory organs, but in *Plp* mutant organs this centriole pair was invariably displaced away from the MT array, and the pair had often separated (arrows, Fig 4C; Fig 4E). This final positioning defect was significantly rescued by the expression of PLP-GFP (Fig 4E). Thus, a centriole pair destined to form the basal body appears to be specified correctly in *Plp* mutant sensory neurons, but it fails to establish and/or maintain its proper position within the neuron, and so cannot organise a cilium.

We wondered whether the basal body in *Plp* mutant sensory organs might be unable to establish and/or maintain its correct position due to defects in the organisation of the organ. We used Serial Block Face-Scanning Electron Microscopy (SBF-SEM) to reconstruct 3 notum bristle sensory organs from 72hr APF WT and *Plp* mutants (a time when organ assembly is complete). In these organs, the neuron (*blue*) forms a single dendrite that extends in a “pore” through the cell body of the sheath cell (*green*), which then attaches to the side of the bristle cell (*magenta*) [41,43]; this organisation was not detectably perturbed in *Plp* mutants (Fig 4F and 4G). Moreover, although the resolution of SBF-SEM images is relatively low, a basal body and transition zone (TZ) were detectable close to the dendritic tip in the 3 WT organs, but not in the dendritic tip of the 3 *Plp* mutant organs (S2 Fig). Taken together, these data suggest that *Plp* mutant sensory organs form normally, but cilia fail to form because the centrioles are not correctly positioned within the neuron.

### In spermatocytes, *Plp* mutant centrioles are mis-oriented and fail to dock properly at the PM, but they can form an axoneme and recruit TZ proteins

**Fig 5:**
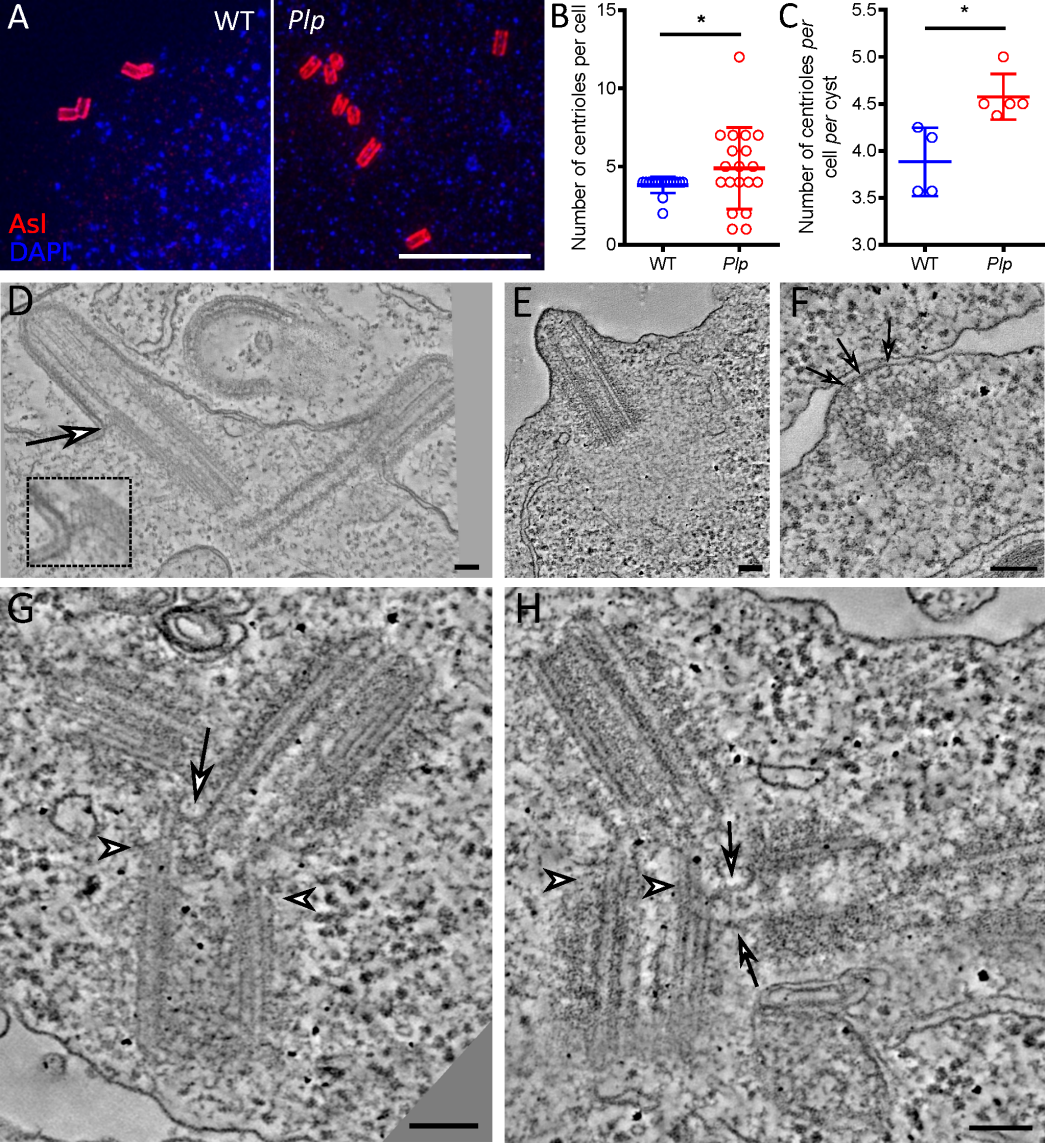
Centrioles and cilia exhibit a variety of defects in *Plp* mutant spermatocytes. (**A**) Images from fixed WT or *Plp* mutant spermatocytes stained with anti-Asl antibodies to reveal the centrioles (*red*) and DAPI to reveal the nuclei (*blue*). (**B,C**) Graphs compare the number of centrioles per cell, or per cell per cyst (a cyst contains the 16 primary spermatocytes derived from the 4 rounds of division of the original spermatocyte gonialblast) in WT or *Plp* mutant testes. This analysis indicates that centrioles tend to overduplicate (as the number of centrioles per cell per cyst increases) but that centrioles can also mis-segregate, leading to individual cells with either too many or too few centrioles. (**D-H**) Micrographs show images from electron tomograms (ETs) of WT (D) or *Plp* mutant (E-H) spermatocytes. Inset and arrows in (D) highlight an electron-dense region that we often observed connecting the centriole/basal body to the plasma membrane at the base of the cilium. Arrows in (F) highlight electron-dense regions that seem to connect the side of this mis-oriented centriole to the plasma membrane (PM). Arrowheads in G and H highlight areas where the centrioles appear to be forming an axoneme-like structure, as the centriole MT triplets transition to axoneme-like doublets; arrows in G and H highlight regions where the clustered centrioles appear to connect to each other. Scale bar = 5μm (A) or 100 nm (E-H). * p < 0.05.

Apart from ciliated sensory neurons, the only other cell type that forms cilia/flagella in *Drosophila* are the cells of the sperm lineage, so we wanted to test if PLP might have similar functions in these cells [42]. The centrioles in primary spermatocytes are longer than in other fly tissues (~1μm compared to ~100-150nm) and they grow short cilia from both mother and daughter centrioles [42,50]. After meiosis, these centrioles will form the basal bodies of the sperm flagellum. WT spermatocytes usually contained 2 centriole pairs, but centriole number was more variable in *Plp* mutant spermatocytes and the centrioles were often short and sometimes appeared fragmented (Fig 5A–5C)—as reported previously [16,47].

An ET analysis revealed that WT centrioles (n = 8) always organised a cilium, and the distal end of the centriole was often connected to the plasma membrane (PM), close to the position where the centriole-MT triplets became axoneme-MT doublets (inset, Fig 5D). In *Plp* mutant spermatocytes we found only a single centriole (1/13) that organised a cilium, and both the centriole and cilium were shorter than normal (Fig 5E). We also observed a single centriole (1/13) making a side-on, rather than end-on, connection to the plasma membrane (Fig 5F). In most cases, however, *Plp* mutant centrioles were found close to the PM, but were not detectably connected to the PM (Fig 5G and 5H). These centrioles were often clustered, and they usually formed a short axoneme-like structure where the centriolar MTs transitioned from triplets to doublets at the presumed distal ends (arrowheads, Fig 5G and 5H). Moreover, they also often appeared to form abnormal proximal-to-distal and distal-to-distal connections to one another (arrows, Fig 5G and 5H), suggesting that these centrioles have prematurely disengaged (and so lost their normal proximal-to-proximal-end connections) but have re-established abnormal connections that allow the centrioles to remain clustered together.

**Fig 6:**
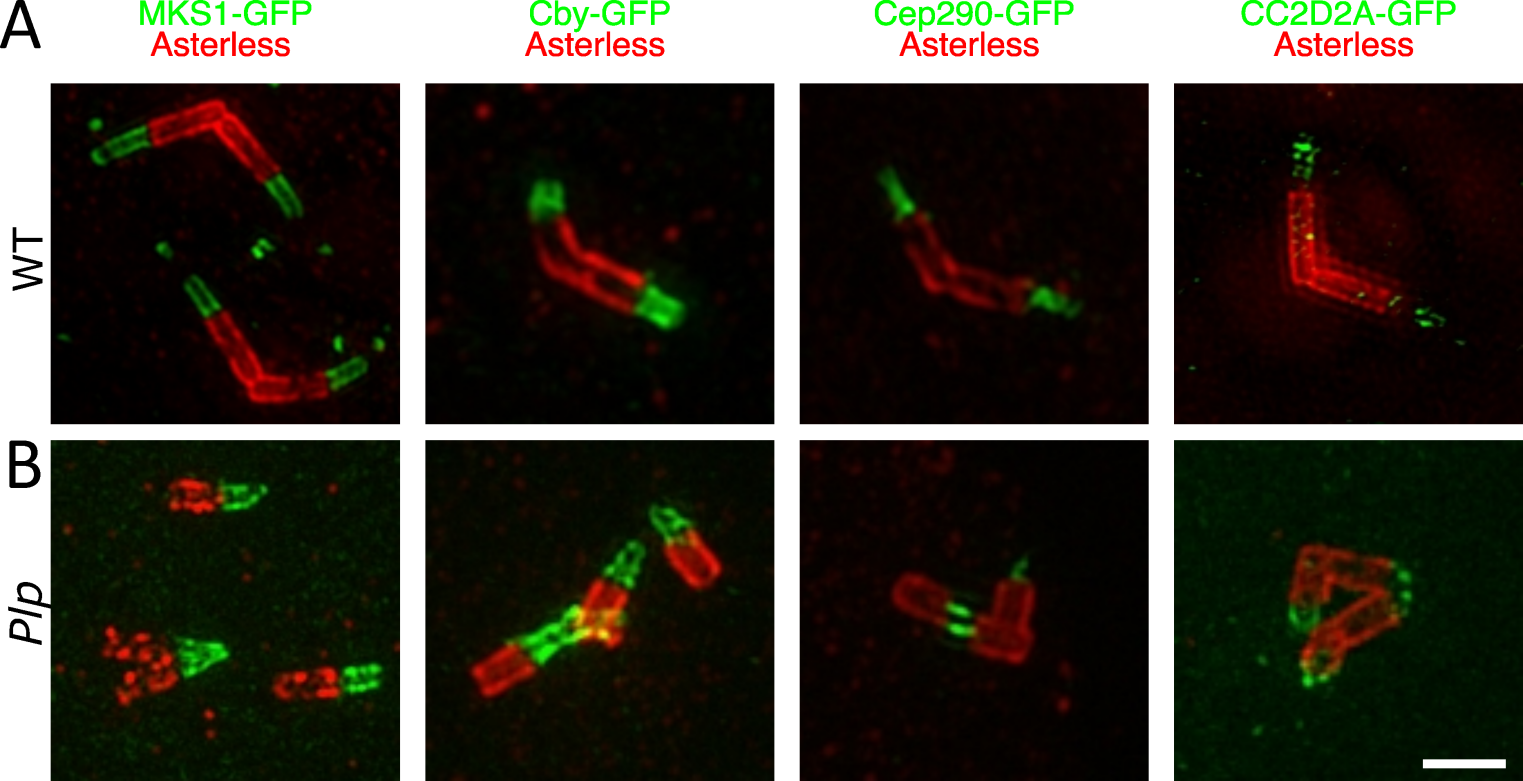
*Plp* mutant centrioles/basal bodies can recruit TZ proteins. (**A,B**) Micrographs show images from fixed WT (A) or *Plp* mutant (B) spermatocytes stained with anti-Asl antibodies to reveal the centrioles (*red*), and anti-GFP antibodies (*green*) to reveal the distribution of GFP-fusions to the TZ proteins MKS1, Cby, Cep290 and CC2D2A (as indicated). Although EM studies show that the vast majority of *Plp* mutant centrioles are not connected to the PM, all of the centrioles appear to organise TZ proteins in a manner that appears to be very similar to the WT centrioles that are forming a cilium. Note that in some instances, the clustering of the centrioles can make this difficult to visualise, as multiple, prematurely separated, centrioles can be clustered one on top of the other, as appears to be the case in the panel showing the localisation of Cby-GFP in the mutant cells (B). Scale bar = 1μm.

As these centrioles seemed to form an axoneme-like structure, we wondered whether they could also recruit TZ proteins. Surprisingly, all of the TZ proteins we examined were strongly recruited to *Plp* mutant centrioles in a manner that appeared to be very similar to that observed in WT centrioles that were forming cilia (Fig 6) [51,52]. Thus, *Plp* mutant centrioles can assemble axoneme- and TZ-like structures even though they are usually not directly associated with the PM.

### PLP helps to organise interphase centriole MTs in spermatocytes

**Fig 7:**
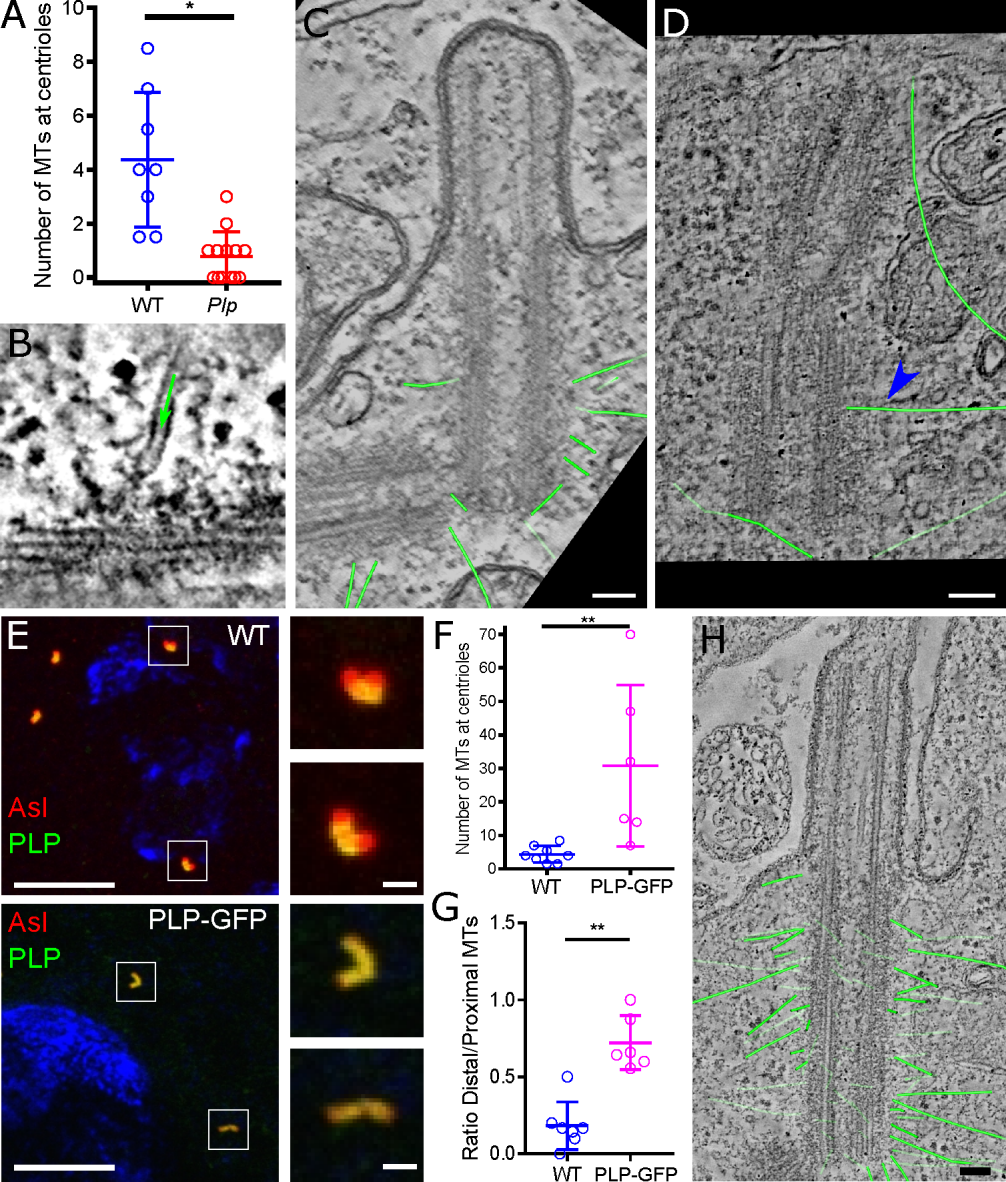
*PLP* helps to recruit MTs to the interphase centriole in spermatocytes. (**A**) Graph quantifies the number of cytoplasmic MTs associated with the interphase centrioles in ETs of WT or *Plp* mutant spermatocytes. (**B-D**) Images from ETs of WT (B,C) or *Plp* mutant (D) spermatocytes: (*green* arrow, B) shows a MT with a “capped” minus end attached to an electron-dense region on the outer wall of a WT centriole; (C,D) shows traces of all the cytoplasmic MTs (highlighted in *green*) associated with WT or *Plp* mutant centrioles. Only one MT is closely associated with the *Plp* mutant centriole (*blue* arrow, D). (**E**) Images show fixed spermatocytes stained with anti-Asl antibodies to reveal the centrioles (*red*) and anti-PLP antibodies (*green*). The DNA is stained with DAPI (*blue*), and the insets illustrate how endogenous PLP is enriched at the proximal ends of the centrioles, while PLP-GFP is distributed more evenly along the entire length of the centriole. **(F,G)** Graphs quantify the number of MTs associated with the interphase centrioles (F), and the ratio of MTs emanating from the distal versus proximal end (G), in WT and PLP-GFP-expressing spermatocytes. **(H)** Image from an ET tracing the centriole associated MTs (*green*) in a spermatocyte expressing PLP-GFP. Scale bar = 100nm (C, D, E) or 10μm (1μm in insets) (E). * p < 0.05, ** p < 0.01.

**Fig 8:**
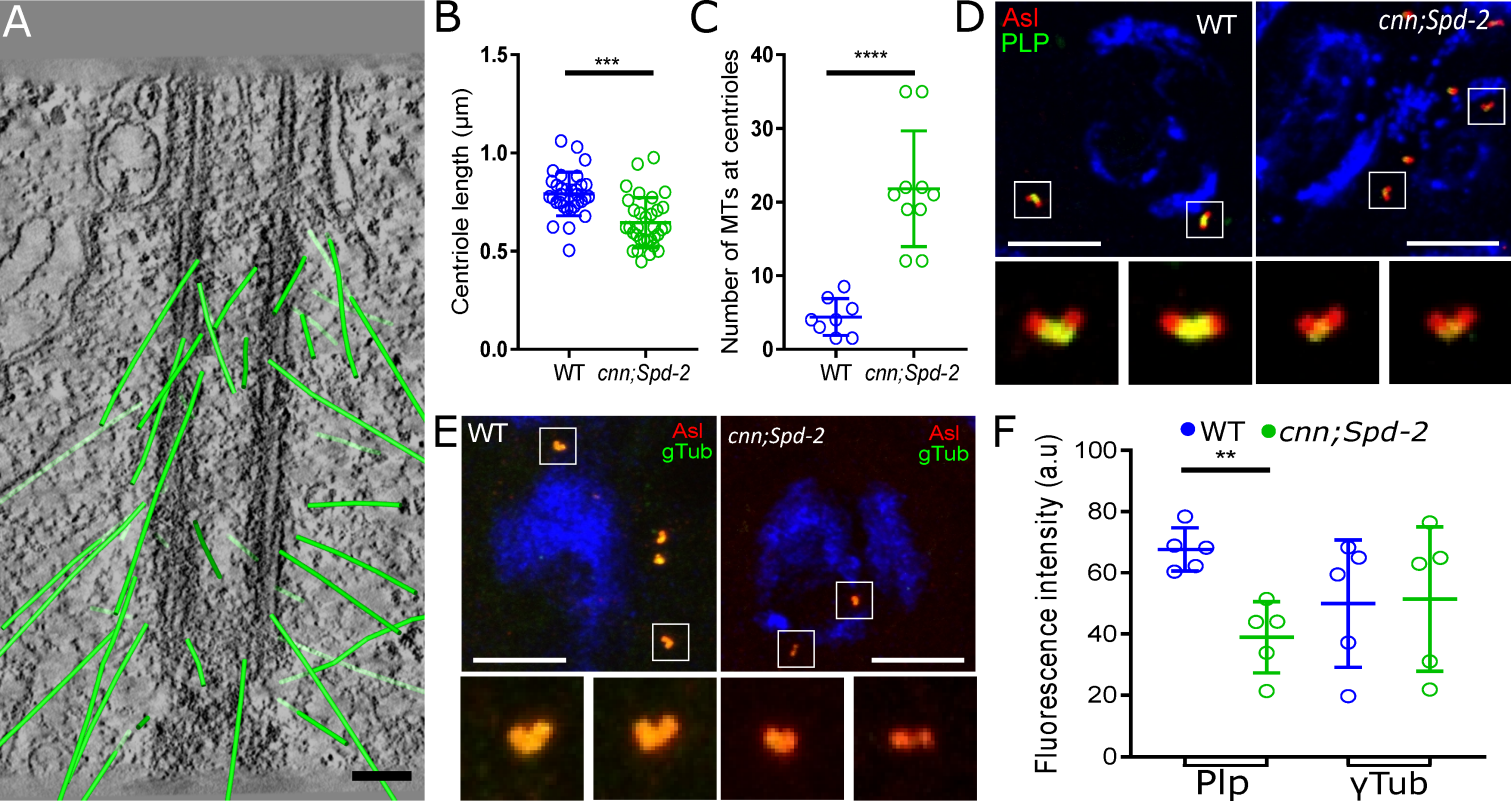
The mitotic PCM organisers Spd-2 and Cnn appear to suppress the MT nucleating capacity of interphase centrioles in spermatocytes. (**A**) Image from an ET tracing the centriole associated MTs (green) in a *cnn*;*Spd-2* double mutant spermatocyte. **(B,C)** Graphs quantify the length of interphase centrioles (B), and the number of MTs associated with the interphase centrioles (C) in *cnn*;*Spd-2* double mutant spermatocytes. (**D-F**) Images show (D,E) and graph quantifies (F) the amount of PLP or **γ**-tubulin (*green*) recruited to interphase centrioles in WT and *cnn*;*Spd-2* double mutant (right) spermatocytes (note that graphs show the levels of these proteins normalised to the amount of the centriole marker Asl, to allow for the centrioles being shorter in the mutant spermatocytes). Scale bar = 100nm (A) or 10μm (D, E). *** p < 0.001, **** p< 0.0001.

EM studies indicated that PLP helps to organise interphase MTs around the wing-disc centrioles, and this was also the case in spermatocytes (Fig 7). WT spermatocyte centrioles typically organised several MTs around themselves, and these usually had their minus ends capped close to the centriole (Fig 7A–7C). In *Plp* mutants the number of MTs associated with the centrioles was reduced, and we only observed a single MT that had its minus end capped at the centriole surface (Fig 7A; arrow, Fig 7D). Endogenous PLP is normally concentrated proximally at spermatocyte centrioles [47] (Fig 8E), and cytoplasmic MTs were preferentially associated with the proximal half of centrioles (Figs 7C and 8F). A PLP-GFP fusion protein expressed from the Ubq-promoter localised along the length of the spermatocyte centrioles (Fig 7G) [47], and this led to an increase in the number of MTs associated with the centrioles (Fig 7G and 7H), and to more of these MTs being associated with the centriole distal end (Fig 7F and 7H). Together, these data strongly suggest that PLP is involved in organising centriole-associated interphase MTs in both spermatocytes and wing-discs.

### The PCM proteins Spd-2 and Cnn help to recruit and/or maintain PLP at interphase centrioles, but antagonise the ability of PLP to organise MTs

How does PLP organise centriole-associated interphase MTs? In cultured cells, PLP recruits key PCM organising proteins such as Cnn and **γ**-tubulin to interphase centrioles [19]. *In vivo*, however, Cnn normally cooperates with Spd-2 to form an expanded scaffold that recruits the *mitotic* PCM [53,54]. We wondered, therefore, whether PLP might promote MT nucleation at interphase centrioles by organising a Spd-2/Cnn scaffold. We therefore assayed several aspects of centriole behaviour in *cnn*;*Spd-2* double mutant spermatocytes (Fig 8). An ET analysis revealed that *cnn*;*Spd-2* double mutant centrioles were shorter than normal but, strikingly, the number of MTs associated with the centrioles was dramatically increased (Fig 8A–8C). Surprisingly, however, the amount of PLP recruited to spermatocyte centrioles was reduced in *cnn*;*Spd-2* double mutants, while the amount of **γ**-tubulin recruited appeared relatively unaffected (Fig 8D–8F). These findings suggest that, in spermatocytes at least, PLP does not help to organise interphase MTs by recruiting the mitotic centrosome scaffolding proteins Cnn and Spd-2.

## Discussion

Here we have used live-cell imaging combined with EM and ET to systematically catalogue the centriole, centrosome and cilium defects in *Plp* mutant pupal notum sensory organs and spermatocytes. We show that WT mother centrioles in interphase are normally surrounded by electron-dense clouds of material that extend outwards from the inner cartwheel spokes. In the absence of PLP, these pericentriolar clouds are greatly diminished. Previous EM studies have identified diffuse “PLP-fibrils” that are organised around the interphase mother centriole in an approximately nine-fold symmetric manner [19]. Thus, PLP is likely to be an important structural component of these pericentriolar clouds. We show that centrioles exhibit multiple and complex defects in the absence of PLP, several of which are in agreement with defects described in previous reports, but several of which have not been reported previously. As we discuss below, we propose that defective pericentriolar cloud assembly could explain most, if not all, of the centriole defects we observe in *Plp* mutants.

The structure of centrioles is subtly perturbed in the absence of PLP: centrioles were too short and we occasionally observed centriole MT doublets that were missing an outer-B-MT. To our knowledge, these observations provide the first evidence that PLP is involved in maintaining centriole structure *per se*, and we suggest that the pericentriolar-clouds, which pass close by the centriolar MTs as they spread outwards from the central cartwheel, may help to establish and/or maintain these B-MTs. It is unclear why centrioles are too short in the absence of PLP, but this could be a result of a general destabilisation of centriole structure.

Pericentrin in vertebrate cells helps to maintain mother/daughter centriole cohesion, and it is cleaved by Separase at the end of mitosis to promote centriole disengagement [13–15]. This has not been reported previously in flies, but we show here that a role in promoting centriole engagement is indeed a conserved feature of Pericentrin/PLP function: centrioles tend to separate prematurely in the absence of PLP and this can lead to centriole overduplication. The pericentriolar clouds appear to directly connect engaged mother and daughter centrioles, and the distance between mothers and their engaged daughters is decreased in *Plp* mutants, as the size of these clouds is reduced. We propose that pericentriolar clouds promote centriole engagement. Importantly, this mechanism cannot be the only one that maintains centriole engagement in flies, as many centrioles still separated at the correct time in our live-cell analysis of SOPs, and we observed engaged mother and daughter centrioles in our EM and ET analysis of *Plp* mutants.

It has previously been shown that PLP recruits PCM components to the interphase centriole in fly cultured cells [19], but the role of PLP in organising MTs around the interphase centirole has not been directly assessed. Our EM analysis of wing-disc and spermatocyte centrioles demonstrates that PLP plays an important part in organising these MTs; the number of interphase centriolar MTs decreased in the absence of PLP (although they were not abolished) and increased when PLP was overexpressed. It is unclear how PLP promotes MT organisation at interphase centrioles, but our data strongly suggests that, in spermatocytes at least, it does not do so by recruiting or regulating Spd-2 and Cnn. These two proteins cooperate to form a scaffold that is essential for mitotic centrosome assembly in flies [53,54], but they are also present at interphase centrioles [19,20], although their role in organsing centrosomal MTs during interphase has not been assessed. Surprisingly, the ability of spermatocyte centrioles to organise MTs during interphase dramatically increased in the absence of Spd-2 and Cnn. A possible explanation for this surprising result is that Spd-2 and Cnn normally interact with PLP at interphase centrioles, but these interactions do not promote MT organisation during interphase, and rather prevent PLP from interacting with other factors that do promote interphase MT organisation. These findings suggest that interphase centrioles can organise MTs independently of key proteins that are required to organise MTs at mitotic centrosomes. Interestingly, in fly embryos, a non-centriolar pool of PLP interacts with Cnn specifically in the peripheral regions of the mitotic PCM, stabilising these outer regions of the Cnn scaffold [17,18,54].

In the absence of PLP, centrioles often fail to migrate properly to the cell cortex, and they fail to establish and/or maintain a proper connection to the PM in cells that form cilia. This presumably explains why *Plp* mutants exhibit severe cilia defects [16]. This is different to the situation in vertebrate cells where cilia lacking Pericentrin are also dysfunctional, but this has been attributed to a failure to properly recruit IFT and Polycystin2 proteins to the cilia [22]. It is unclear why centrioles fail to establish and or maintain a connection to the PM in *Plp* mutant flies. One possibility is that an inability to properly organise interphase MTs may contribute to the inability of the mutant centrioles to migrate properly to the apical cortex and to establish and/or maintain a connection with the PM. Alternatively, the pericentriolar clouds could themselves interact with the PM and so help to establish and/or maintain the proper cortical positioning of the centrioles.

Finally, our analysis of centriole behaviour in *Plp* mutant spermatocytes revealed that even when these centrioles fail to connect properly to the PM, they can still assemble an axoneme-like structure that can recruit TZ proteins. The ability to recruit and organise TZ proteins is very surprising, as many of these proteins contain domains that are thought to be membrane-associated, yet the centrioles in *Plp* mutants are not connected to the PM. Thus, centrioles that are destined to form basal bodies can at least partially organise an axoneme and a TZ even when they fail to dock at the PM.

## Materials and methods

### Fly stocks

*w67* was used as a WT control in all experiments. Two previously described *Plp* mutant alleles were used in this study: *plp*^*2172*^ (a P-element insertion in a an intron between exon 6 and 7 of the 16 exons of the longest transcript) and *plp*^*5*^ (a Q1900STOP nonsense mutation that results in the expression of a truncated protein that lacks the C-terminal ~900aa that encode the centriole-targeting PACT-domain of Plp); both alleles appear to behave as functional nulls [16–18,47], although the Plp gene is large and encodes many transcripts, so we cannot be certain these alleles are true functional nulls. Both alleles produced indistinguishable phenotypes and were used interchangeably. The *cnn;Spd-2* double mutant stock was created with the following lines: *cnn*^*hk21*^ [55] and *cnn*^*f04547*^ [56]; *Spd-2*^*z3-5711*^ and *Df(3L)st-j7* (Bloomington stock #5416) [57]. UASt-GFP-Asterless (JWR and Renata Basto) driven by the Scabrous–Gal4 transgene, a pan-neuronal Drosophila driver [58] was used to mark the centrioles in the SOP lineage. The following transgenic lines were described previously: MKS1-GFP and CC2D2A-GFP [52], Cby-GFP [59], Cep290-GFP [60], Polo-GFP [61] and PLP-GFP [47].

### Antibodies

The following primary antibodies were used: Guinea-Pig anti-Asterless [62] (RaffLabDB#191); Rabbit anti-PLP [16] (RaffLabDB#86), Rabbit anti-Cnn [56] (RaffLabDB#37), Mouse anti-γTub (GTU88, Sigma, cat. T6557), Rabbit Anti-Spd-2 [63] (RaffLabDB#57). The following secondary antibodies were used: Anti-Guinea Pig IgG Alexa Fluor 568 (Invitrogen, cat. A11075), Anti-Guinea Pig IgG Alexa Fluor 633 (Invitrogen, cat. A21105), Anti-Rabbit IgG Alexa Fluor 488 (Invitrogen, cat. A21206), Anti-Mouse IgG Alexa Fluor 488 (Invitrogen, cat. A11001) and Anti-Mouse IgG Alexa Fluor 568 (Invitrogen, cat. A11004).

### Fluorescence microscopy

3D-SIM of testis was performed as described previously [46]. Live imaging of pupae centrioles and MTs was performed in a Nikon Eclipse TE200-E spinning disk confocal system, equipped with an EM-CCD Andor iXon+ camera, controlled by the Andor IQ2 software. Pupae were prepared for imaging as previously described [45]. Testis squashes of whole testis and spermatocyte cysts were performed as previously reported [46,63]. Immuno-fluorescence images were acquired in an Olympus Fluoview FV-1000 microscope and software. Each slide imaged had both a control and a mutant testis in opposite sides of the slide to guarantee equal staining. Imaging conditions were maintained between slides.

### Image analysis

4D centriole tracking was performed in 8-bit converted images with Fiji [64] using the Trackmate plugin [65] using the following parameters: detection with sub-pixel localization using LOG method, threshold of 200 and estimated blob diameter set to 0.9μm. No initial threshold was applied to detections. The simple LAP tracker was used to create the tracks. Both linking max distance and gap-closing max distance were set to 2μm and Gap-closing max frame gap was set to 2. Centriole positions in 4D were extracted from Trackmate and exported into Prism (Graphpad) for analysis and plotting. Angles of division were calculated using the line function of Fiji and measured in relation to the anterior to posterior axis. Circular plots were computed using the circular package in Rstudio. Immuno-fluorescence images were analysed in Fiji, using a purpose-written macro (available on request) to automatically segment and extract the mean sum of marker fluorescence per centriole. Asterless staining was presented as mean sum per centriole, while all other markers where presented as ratio (marker mean sum)/(Asterless mean sum) per centriole. All data analysis and plotting was performed with Prism (Graphpad).

### Electron microscopy, tomography and Serial Block Face Scanning Electron Microscopy

Samples, processing and modelling of electron microscopy and tomography data of testis centrioles and pupal samples was performed as previously published [46,52]. Data was automatically acquired in an FEITecnai T12 at 120KV using a Gatan OneView digital camera with the Navigator function of SerialEM software [66,67]. For SBFSEM samples were prepared according to [68] with some modifications. Briefly, samples were fixed overnight in 2.5% glutaraldehyde, 4% Paraformaldehyde and 0.1% tannic acid at 4°C (from a freshly prepared 10% stock) in 0.1M PIPES buffer, pH 7.2. Samples were then washes twice for 30min in 0.1M PIPES, followed by a 30 min wash in 50mM glycine in 0.1M PIPES to quench free aldehydes, and another 30 min wash in 0.1M PIPES. Samples were then embedded in 4% low melting point agarose plus 4% porcine gelatin (Melford, cat. L1204). Small cubes of agarose with one pupae each were then further fixed in 1.5% potassium ferricyanide and 2% osmium tetroxide in 0.1M PIPES for 1 hour at 4°C. Samples were then washed three times for 10 min in water. Next samples were incubated in thiocarbohydrazide for 20min at room temperature, followed by three 10min washes in MQ water. Samples were then incubated in 2% osmium tetroxide in MQ water for 30min at 4C. After three 10 min MQ washes, samples were incubated in 1% uranyl acetate in MQ water overnight at 4°C. Samples were washed three times for 10 min in MQ water followed by *en-bloc* staining with lead aspartate solution for 30 min at room temperature. After three 10 min washes in MQ water, dehydration was performed in ice with pre-cooled solutions of 30%, 50%, 70%, 90%, 100%, 100% anhydrous ethanol for 10 min each, followed by ice-cold acetone for 10 min and a further 10 min in acetone at room temperature. Embedding was performed in acetone:Durcupan resin (Sigma cat. 44610) mix of 25% for 3 hours, 50% overnight, 75% for 3 hours and four times 100% Durcupan resin freshly prepared for 8–14 hours each. Samples were embedded in Beem capsules and cured at 60°C for 48–72 hours. Samples were mounted onto 3View pins using conductive silver epoxy and volumes acquired in a Zeiss Merlin Compact VP FEG-SEM equipped with Gatan 3View microscope. Sections were acquired using Digital Micrograph 2.0 every 50 nm, at 3.5KeV with 30 m aperture and VP set to 50 Pa. Images were aligned and modelled using the software package IMOD [66].

## Supporting information

S1 Fig *Plp* mutant pupal notum centrioles have reduced pericentriolar clouds. (**A,B**) Images from ETs of WT (A) or *Plp* mutant (B) centrioles in pupal notum cells. **(A’,B’)** Images are the same as in (A,B) but with the electron dense pericentriolar clouds highlighted in *green*. Although centriole ultrastructure in the pupal notum is difficult to discern (presumably because the cuticle in this tissue makes fixation difficult), the pericentriolar clouds clearly appear to be reduced in the *Plp* mutant tissue. Scale bar = 100 nm.
(PDF)

S2 Fig *Plp* mutant sensory cilia lack centrioles. **(A,B)** Images from an SBF-SEM analysis of WT (A) or *Plp* mutant (B) Sensory Organs (with the neuronal cell outlined by *green dotted-line*) showing the ciliary invagination (outlined by *blue dotted-line*). Although the resolution of these images is low, the basal body can be observed at the base of the WT cilium (arrowhead), and the TZ can be seen as an electron-dense constriction of the PM just above the basal body. A ciliary invagination is present in the *Plp* mutant neuron, but no centriole or TZ structures are detectable. Scale bar = 2μm.
(PDF)

S1 Table Numbers analysed and biological repeats. The number of samples analysed and the number of biological repeats for all experiments described throughout the manuscript.
(PDF)
