# Supplementary figures and images for: Drosophila PLP assembles pericentriolar clouds that promote centriole stability, cohesion and MT nucleation
Source: PLoS Genet. 2018 Feb 9;14(2):e1007198. doi: 10.1371/journal.pgen.1007198 (PMC5823460; doi:10.1371/journal.pgen.1007198)

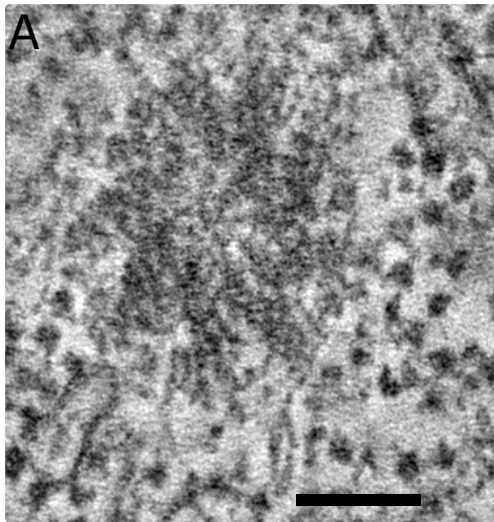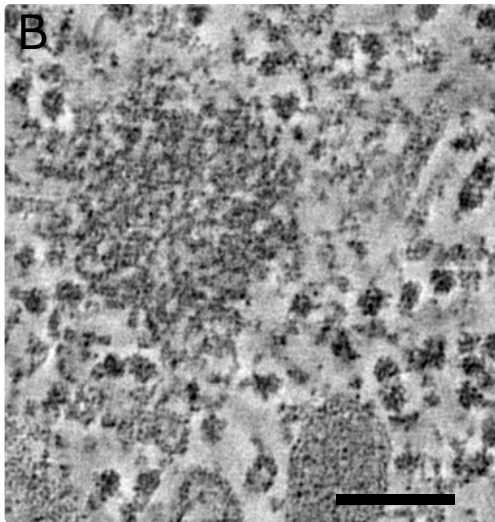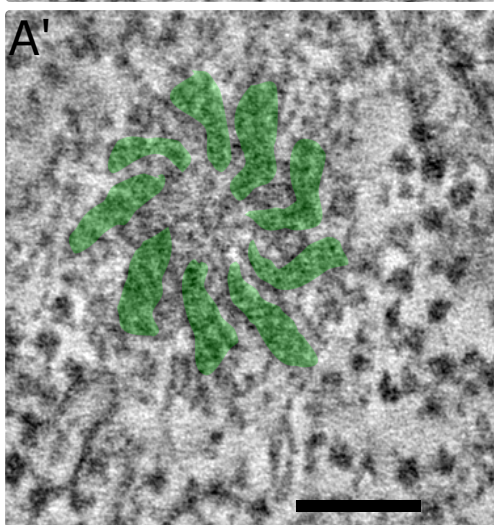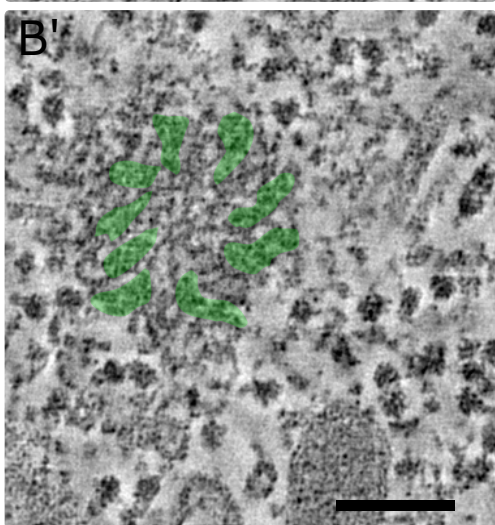

Supplement: S1 Fig — (A,B) Images from ETs of WT (A) or Plp mutant (B) centrioles in pupal notum cells. (A’,B’) Images are the same as in (A,B) but with the electron dense pericentriolar clouds highlighted in green. Although centriole ultrastructure in the pupal notum is difficult to discern (presumably because the cuticle in this tissue makes fixation difficult), the pericentriolar clouds clearly appear to be reduced in the Plp mutant tissue. Scale bar = 100 nm. (PDF) [file pgen.1007198.s001.pdf]

A

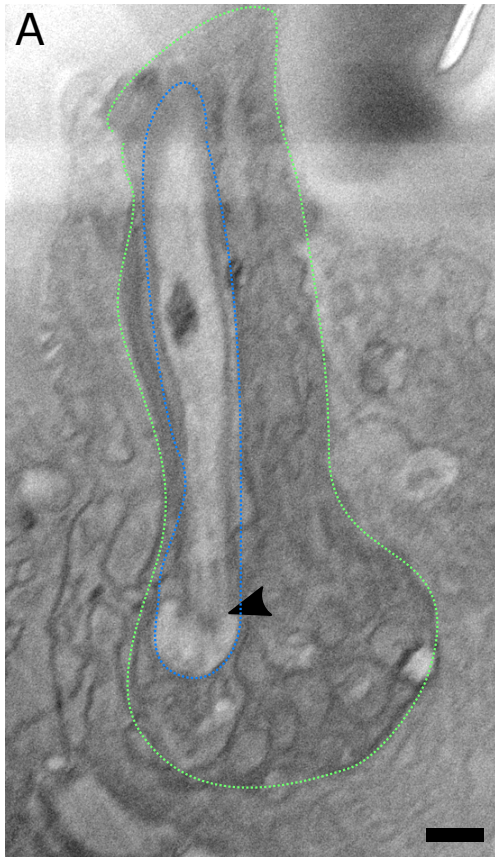

B

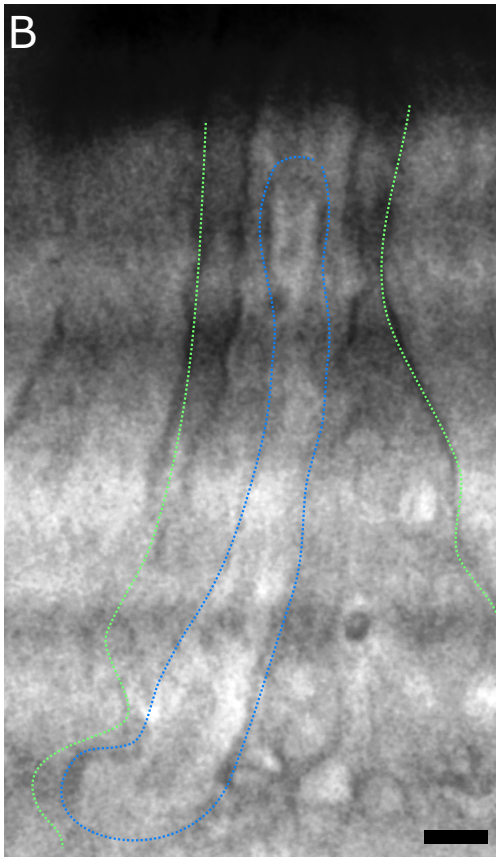

Supplement: S2 Fig — (A,B) Images from an SBF-SEM analysis of WT (A) or Plp mutant (B) Sensory Organs (with the neuronal cell outlined by green dotted-line) showing the ciliary invagination (outlined by blue dotted-line). Although the resolution of these images is low, the basal body can be observed at the base of the WT cilium (arrowhead), and the TZ can be seen as an electron-dense constriction of the PM just above the basal body. A ciliary invagination is present in the Plp mutant neuron, but no centriole or TZ structures are detectable. Scale bar = 2μm. (PDF) [file pgen.1007198.s002.pdf]
