# Supplementary material for: Drosophila PLP assembles pericentriolar clouds that promote centriole stability, cohesion and MT nucleation
Source: PLoS Genet. 2018 Feb 9;14(2):e1007198. doi: 10.1371/journal.pgen.1007198 (PMC5823460; doi:10.1371/journal.pgen.1007198)
Supplement: S1 Table — The number of samples analysed and the number of biological repeats for all experiments described throughout the manuscript. (PDF) [file pgen.1007198.s003.pdf]

Table S1: Numbers analysed and biological repeats

Figure - Panel	Numbers analysed	Biological repeats
**1, 2**	SOPs first divisions: 15 WT, 17 Plp	5 pupae per genotype
**2-C**	Centrioles: 19 per genotype	
**2-E**	Centrioles: 8 WT, 14 Plp	Different pupae: 3 per genotype
**3-D**	Centrioles (minimum of 3 clouds per centriole): 16 WT, 17 Plp, 12 PLP-GFP	Different wingdiscs form different flies: 4 for WT and Plp and 2 for PLP-GFP
**3-E**	Centriole pairs: 17 WT, 13 Plp, 9 PLP-GFP	
**3-F**	Centrioles: 27 WT, 36 Plp, 24 PLP-GFP	
**3-H**	Centrioles: 40 WT, 39 Plp, 13 PLP-GFP	
**4-E**	45 sensory organs per genotype	Pupae: 7 WT, 8 Plp, 7 PLP-GFP
**5-B**	At least 30 cells per squashes	Testis squashes of different flies: 18 WT, 19 Plp
**5-C**	Testis cysts squashes form different flies: 4 WT, 5 Plp	
**5-D**	Centrioles: 8 WT	Different testis from different flies: 3 per genotype
**5-E to 5-H**	Centrioles: 13 Plp	
**6-A, 6-B**	> 30 centriole pairs analysed per labelled per genotype	Different testis from different flies: 3 per genotype
**7-A**	Centrioles: 8 WT, 13 Plp	Different testis from different flies: 3 per genotype
**7-F**	Centrioles: 7 WT, 6 PLP-GFP	
**7-G**	Centrioles: 8 WT, 6 PLP-GFP	
**8-A**	At least 30 cells per biological repeats were analysed for all genotypes	Different testis from different flies: 3 per genotype
**8-B**	Centrioles: 32 WT and 33 cnn; Spd-2	Different testis squashes from different flies performed in parallel for WT and mutant: 7 Asl, 5 Spd2, 4 Cnn, 7 γTub, 6 Polo-GFP
**8-D**	At least 30 cells per biological repeats were analysed for all genotypes	Different testis squashes from different flies performed in parallel: 5 per genotype
**8-F**	Centrioles: 8 WT, 10 cnn; Spd-2	
